# Ferroptosis: Underlying mechanism and the crosstalk with other modes of neuronal death after intracerebral hemorrhage

**DOI:** 10.3389/fncel.2023.1080344

**Published:** 2023-02-06

**Authors:** Yuan Cao, Wenbiao Xiao, Shuzhen Liu, Yi Zeng

**Affiliations:** ^1^Department of Geriatrics, The Second Xiangya Hospital of Central South University, Changsha, Hunan, China; ^2^Department of Radiology, The Second Xiangya Hospital of Central South University, Changsha, Hunan, China

**Keywords:** intracerebral hemorrhage, ferroptosis, apoptosis, necroptosis, autophagy

## Abstract

Intracerebral hemorrhage (ICH) is a serious cerebrovascular disease with high rates of morbidity, mortality, and disability. Optimal treatment of ICH is a major clinical challenge, as the underlying mechanisms remain unclear. Ferroptosis, a newly identified form of non-apoptotic programmed cell death, is characterized by the iron-induced accumulation of lipid reactive oxygen species (ROS), leading to intracellular oxidative stress. Lipid ROS causes damage to nucleic acids, proteins, and cell membranes, eventually resulting in ferroptosis. In the past 10 years, ferroptosis has resulted in plenty of discoveries and breakthroughs in cancer, neurodegeneration, and other diseases. Some studies have also reported that ferroptosis does occur after ICH *in vitro* and *in vivo* and contribute to neuronal death. However, the studies on ferroptosis following ICH are still in the preliminary stage. In this review, we will summarize the current evidence on the mechanism underlying ferroptosis after ICH. And review the traditional modes of neuronal death to identify the crosstalk with ferroptosis in ICH, including apoptosis, necroptosis, and autophagy. Additionally, we also aim to explore the promising therapeutic application of ferroptosis in cell death-based ICH.

## 1. Introduction

Intracerebral hemorrhage constitutes 10–15% of all strokes but accounts for almost 50% of stroke mortality worldwide (Thrift et al., 2017). For patients with ICH, the rupture of blood vessels in the brain results in primary brain injury and secondary brain injury (SBI) (Qureshi et al., 2009). These patients suffer from a lack of effective treatments to overcome harmful brain symptoms and research efforts lag behind those for ischemic stroke (Donnan et al., 2010; Hemphill et al., 2015). In general, it is thought that the main mechanisms of neuronal death in ICH are excitotoxicity, the toxicity of blood, oxidative stress, mitochondrial death pathways, the release of free radicals, protein misfolding, apoptosis, necroptosis, necrosis, autophagy, and inflammation (Wang, 2010; Aronowski and Zhao, 2011). Such mechanisms occur around the hematoma and in remote areas of the brain, not necessarily in contact with the bleeding. These all lead to neuronal death and dissipation of function which are particularly crucial because adult neurons have a limited ability to proliferate or replace. In the past, common neuronal death modalities following ICH included apoptosis, necroptosis, pyroptosis, autophagy, and parthanatos (Zhang et al., 2022c). Until, Li et al. (2017b) confirmed the occurrence of ferroptosis through the ICH mouse model, which was the earliest report of neuronal ferroptosis after ICH. They also showed that ferrostatin-1 (Fer-1), a ferroptosis inhibitor, improved the neurological functions of mice after acute ICH (Li et al., 2017b). In Zille et al. (2017) reported that necroptosis and ferroptosis inhibitors each abrogated neuronal death by >80% after ICH and had similar therapeutic windows *in vitro*. So, Ferroptosis may provide new insights into neuronal death after ICH. Subsequently, many researchers have investigated the mechanism of ferroptosis in ICH, intending to identify new directions and targets for treating SBI after ICH (Li et al., 2018, 2020; Bao et al., 2020). Multiple modes of cell death after ICH have been identified. However, the crosstalk between cell death post-ICH is ambiguous, which makes it difficult for scientific researchers to explore the prevention and treatment of ICH (Fricker et al., 2018). In this review, we specifically focus on the mechanism of ferroptosis in neuronal death after ICH and compare the similarities and differences between ferroptosis and several dominant modes of neuronal death, such as apoptosis, necroptosis, and autophagy which exactly can be observed in the pathogenesis of ICH. Additionally, inhibiting neuronal death is a critical component of future therapeutic strategies for ICH. we also search for promising therapeutic applications to improve nerve function after ICH.

## 2. Ferroptosis in ICH

Ferroptosis, a newly identified iron-dependent concept of regulated cell death (RCD) type, was first proposed by Dixon et al. (2012). It is associated with iron, amino acid, and lipid metabolism. The iron-dependent accumulation of lipid peroxidation is the key trigger (Dixon et al., 2014; Conrad et al., 2018; Fujii et al., 2020). The Nomenclature Committee on Cell Death (NCCD) defined ferroptosis as “a form of RCD initiated by oxidative perturbations of the intracellular microenvironment that is under constitutive control by glutathione peroxidase 4 (GPX4) and can be inhibited by iron chelators and lipophilic antioxidants” (Galluzzi et al., 2018). During intracerebral hemorrhage, there is a high flow of iron originating from hemoglobin and hemoglobin, which contributes to cell death that may occur hours or days after the bleeding, and other factors released from blood may also play a role. In Zille et al. (2017), reported that the ICH model treated with Hb had an increased level of extracellular regulated protein kinases (ERK1/2). ERK1/2 is a critical signal in the RAS-RAF-MEK pathway in the process of ferroptosis providing sufficient evidence for the occurrence of neuronal ferroptosis after ICH. In another study, Zhang et al. (2018) showed that the expression of GPX4 was markedly reduced during acute ICH. GPX4 is an important antioxidant that protects neurons against oxidative stress and ferroptosis. Many studies have also revealed that the administration of ferrostatin-1 (Fer-1), a specific inhibitor of ferroptosis, prevented neuronal death and improved neurological function after ICH *in vitro* and *in vivo* (Wu et al., 2011; Li et al., 2017b; Stokum et al., 2020). Li et al. (2017b) also used transmission electron microscopy to find mitochondrial morphological atrophy characteristic of ferroptosis in perihematoma neuronal cells, which may provide strong evidence for the occurrence of ferroptosis after intracerebral hemorrhage. These findings fill an important gap in ferroptosis after ICH and provide a vital foundation for cell death-based ICH treatment in the future.

## 3. The underlying mechanisms of ferroptosis in ICH

**FIGURE 1:**
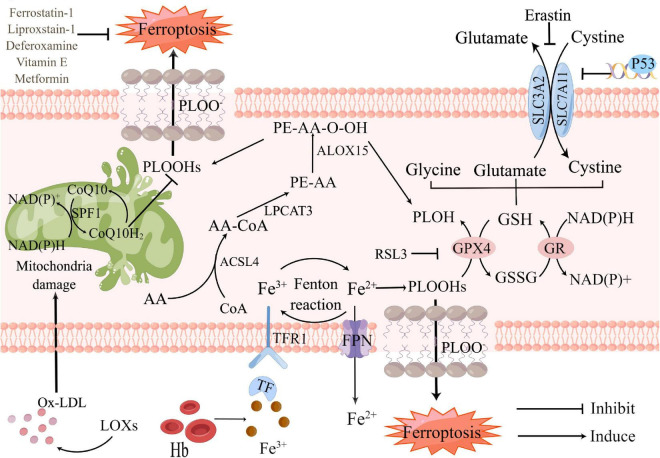
Core mechanisms and signaling pathways of ferroptosis in Intracerebral hemorrhage (ICH). Ferroptosis is driven by the accumulation of PLOOHs, which is tightly linked to the metabolism of amino acids, iron, and lipids. Iron overload and antioxidants lacking as the major component of lipid peroxidation, which contribute to ferroptosis. Fe3+, released from hemoglobin after ICH, converts into Fe2+ by Fenton reaction. Too much Fe2+ causes iron overload in neurons, accelerating intracellular reactive oxygen species (ROS) production and promoting ferroptosis. GSH-GPX4 and FSP1-CoQ are the two main pathways against ferroptosis. Restriction of either pathway will promote ferroptosis. PLOH, phospholipid alcohol; PLOOHs, phospholipid hydroperoxides; FSP1, ferroptosis suppressor protein 1; CoQ10, coenzyme Q10; CoQ10H2, ubiquinol-10; Ox-LDL, oxidized low-density lipoprotein; LOXs, lipoxygenases; Hb, hemoglobin; Fe3+, ferric iron; Fe2+, ferrous iron; ACSL4, Acyl-CoA synthase long-chain family member 4; AA-CoA, arachidonoyl-CoA; LPCAT3, lysophosphatidylcholine acyltransferase 3; FPN, ferroportin; RSL3, RAS-selective lethal 3; GPX4, glutathione peroxidase 4; GSH, glutathione; GSSG, glutathione disulfide; GR, glutathione reductase; ALOX15, arachidonate 15-lipoxygenase; SLC3A2, solute carrier family 3 member 2; SLC7A11, solute carrier family 7 member 11.

So far, the metabolic mechanisms of ferroptosis after ICH seems to be tightly linked to three main categories: the metabolism of amino acids, iron, and lipids, which involve a complex network to shape oxidative stress (Figure 1). Metabolic dysregulation of any one of them may influence ferroptosis. Any molecular change or pharmacological intervention that regulates any of these elements may affect the final consequences of ferroptosis (Liu and Gu, 2022). Strategies targeting ferroptosis pathways have resulted in neuroprotection in preclinical models and some of these have shown promise for patients with ICH (Stockwell et al., 2017). Understanding the mechanisms of ferroptosis after ICH will provide a vital foundation for cell death-based ICH treatment and diagnosis.

### 3.1. Amino acid metabolic pathway

Amino acid metabolism is tightly linked to the regulation of ferroptosis (Angeli et al., 2017). Upregulating GPX4 expression in the ICH model can inhibit ferroptosis and treat ICH (Peng et al., 2022). The GPX4 is currently recognized as a central repressor of ferroptosis, and its activity depends on antioxidant glutathione (GSH). The GSH is a tripeptide composed of glutamic acid, cysteine, and glycine. The three kinds of amino acids are from different pathways. The system xc- antiporter, comprised of SLC7A11 and SLC3A2, is responsible for the transmembrane import of extracellular cystine, which is reduced back to intracellular cysteine. Due to the limited concentration of cysteine in cells, cysteine is considered to be the rate-limiting precursor for GSH synthesis. Glutamate and glutamine are also important regulators of ferroptosis (Gao et al., 2015). Researchers have found in mice, rabbits, and patients with ICH that glutamate levels in brain tissue surrounding the hematoma were elevated (Li et al., 2017b; Epping et al., 2022). The addition of human glutamate to the culture medium of HT22 hippocampal neurons resulted in a significant increase in cell death (Chen et al., 2022a). Li et al. (2017b) found that the application of glutaminase inhibitors could inhibit the decomposition of glutamine into glutamate, and significantly reduce the number of degenerate nerve cells around hematoma. These all confirmed that poor clinical outcomes and increased volume of the residual cavity after ICH are associated with high concentrations of glutamate in blood within the first 24 h from symptom onset (Li et al., 2017a). The presence of large amounts of glutamate will be the rate-limiting precursor for GSH synthesis which is the key to ferroptosis (Leasure et al., 2021). Some study has also highlighted the role that selenium plays in modulating ferroptosis *via* its co-translational incorporation into selenocysteine in GPX4 (Ingold et al., 2018). A single dose of Se delivered into the brain drives antioxidant GPX4 expression, protects neurons, and improves behavior in an intracerebral hemorrhage model. These all findings give us some insights into the treatment of ICH by inhibiting ferroptosis based on amino acid metabolism.

### 3.2. Iron metabolism

Iron metabolism disorder is thought to be a key factor in ferroptosis. While lipid peroxidation causes ferroptosis, an increase in intracellular iron is a risk factor for ferroptosis, and the level of damage is greater when iron is raised. Iron, a putative neurotoxin, is a major product of lysed erythrocytes in hematoma after ICH. It can be engulfed by microglia and infiltrating macrophages in the perihematomal zone and metabolized into ferrous/ferric iron, which induces the formation of lethal ROS and lipid peroxidation contributing to ferroptosis and SBI (Zhang et al., 2022a). Since intraparenchymal hematomas and red blood cells are the main sources of free iron in the ICH brain, resolution of hematoma and the clearance and phagocytosis of red blood cells might reduce iron-induced ferroptosis. Further research is necessary to examine these methods for treating iron-induced ferroptosis. Deferasirox (DFR), a trivalent iron chelator, suppressed microglia/macrophage activation in peri-hematoma area at 3 days after ICH and significantly suppressed the intracellular Fe2+ accumulation and cell death caused by hemin exposure. It might be a useful therapeutic agent for the therapy of ICH (Imai et al., 2021). Excessive iron form highly toxic hydroxyl radicals and trigger ROS formation to attack DNA, proteins, and lipid membranes, thereby disrupting cellular functions and causing neuronal death (Magtanong and Dixon, 2018; Zhang et al., 2022c). Wu et al. have shown that iron ions overload occurs in the posterior brain of ICH which accumulates within 3 days after ICH causing brain edema and cell death (Urday et al., 2015; Selim, 2022). Mechanistically, following ICH, excessive ferric irons from red blood cells (RBC) bind to transferrin (TF) in serum transported into cells through receptor-mediated effects (Schwartz-Duval and Sokolov, 2022). Ferric irons are reduced to ferrous ions by divalent metal transporter 1 (DMT1) and accumulation in nerve cells. Ferrous ions induce excessive lethal ROS and lipid peroxide formation. In a previous study of ICH in mice, two kinds of iron chelators, deferoxamine (DFX) and VK28, reduced the number of nerve cell death, iron ion accumulation, microglia activation and improved the neural function of mice eventually (Li et al., 2017a). Iron-dependent Fenton chain reaction is likely the key to ferroptosis. When GPX4 is lacking, phospholipid hydroperoxides (PLOOHs) in the cell cannot be removed in time and will react with iron to trigger the Fenton chain reaction, generating more PLOOHs. This is also a hallmark of ferroptosis (Conrad and Pratt, 2019). This reaction not only damages lipids and proteins but also causes oxidative damage to DNA, including DNA base modifications and DNA strand breaks (Gu et al., 2022; Wu et al., 2022). While an iron overload does not always induce ferroptosis, it will enhance cell death when it occurs. The mechanisms of brain iron metabolism are still poorly understood, which greatly limits the development of therapeutic drugs targeting brain iron efflux after ICH. Therefore, elucidating the mechanisms underlying iron metabolism is crucial to developing effective therapeutic strategies to reduce iron accumulation in ICH (Bao et al., 2020).

### 3.3. Lipid metabolism

In the physiological state, the dynamic balance between oxidation and antioxidant reactions helps to keep the body operating normally. Polyunsaturated fatty acids (PUFAs), an integral component of the plasma membrane, may be oxidized *in vivo* enzymatically. Excess oxidized PUFA is converted by GPX4 to a non-toxic form. PUFAs can also be generated with fenton chemistry but a functional GPX4/GSH axis should be able to maintain homeostasis. One of the features of ferroptosis is the accumulation of LPO which causes a variety of damage to the structure and function of cells and membranes (Dixon et al., 2012). LPO is a lipid with a peroxide group formed after the reaction of unsaturated fatty acid chains with free radicals or ROS. Under normal conditions, the level of LPO is extremely low, but in pathological conditions, increased lipid peroxidation can lead to an increase. It has been illuminated as a clear mechanism to produce highly LPO with ROS up-regulation through the fenton chemistry (Liang et al., 2019; Lei et al., 2020). After ICH, ferrous ions overload, GPX4 deficiency, and PLOOHs cannot be cleared in time pointing to the susceptibility of the fenton chemistry. Overload ROS that exceeds the antioxidant capacity of cells leads to an enhanced oxidative stress response, which directly or indirectly damages proteins, nucleic acids, lipids, and other macromolecular substances (Tan et al., 2022). Finally, the membrane is damaged and the cell collapses and dies due to the lipid peroxidation inside the phospholipid of the cell membrane. In addition, GPX4, a selenoprotein, implies that selenium availability impacts the sensitivity to ferroptosis. It functions to reduce PLOOHs to lipid alcohols (L-OH) and to reduce H_2_O_2_ to H_2_O then reduce the damage to membrane function (Tang et al., 2019). Delivery of selenium to cells or animals to upgrade GPX4 level can suppress ferroptosis, including in a mouse model of ICH (Friedmann Angeli and Conrad, 2018; Ingold et al., 2018; Alim et al., 2019). However, abnormal amino acid metabolism after ICH results in GPX4 deficiency as mentioned above. Other research has shown that the suppression of GPX4 is related to cyclooxygenase-2 (COX-2) and the increased expression of 15-lipoxygenase (ALOX15) (Meng et al., 2022). Li et al. (2017b) observed in a collagenase-induced ICH model that COX-2, encoded by-product cyclooxygenase-2 (PTGDS-2), was highly expressed in post-ICH neurons. High expression of COX-2 contributes to ferroptosis by inhibiting the antioxidant effect of GPX4. In addition, ALOX15 participates in the programmed degradation of organelles by binding to the membranes of various organelles in cells. *In vitro* ALOX15 is found to bind to mitochondria leading to membrane disintegration and ROS production (Choudhary et al., 2022). Currently, increased ALOX was observed after ICH in both humans and mice (Karuppagounder et al., 2018). Lipoxygenases (LOXs) have been also implicated as central players in ferroptosis (Shah et al., 2018). 5-lipoxygenase (5-LOX) inhibitor Zileuton could inhibit ferroptosis and play a protective role in nerve cells through the reduction of lipid peroxides (LPO) production (Gao et al., 2015; Shah et al., 2018). Therefore, the regulation of enzymes in lipid metabolism and enhancement of cellular antioxidant effects are other potential targets for inhibiting ferroptosis.

## 4. The crosstalk between ferroptosis and other traditional cell death pathways in ICH

There are various forms of cell death have been identified in ICH earlier except for ferroptosis (Chen et al., 2012; Zille et al., 2017; Zhang et al., 2018, 2020; Djulbegovic and Uversky, 2019), including apoptosis (de Oliveira Manoel, 2020; Gan et al., 2021; Tarantini et al., 2021; Grootaert and Bennett, 2022; Kuramoto et al., 2022), necroptosis (Yuan et al., 2019; Meng et al., 2021), autophagy (Chen et al., 2012; Cao and Mu, 2021; Zhang et al., 2022b) and so on in humans and experimental animals. Ferroptosis is mainly characterized by lipid peroxidation-induced cell death, which is morphologically, biochemically, and genetically distinct from apoptosis, necroptosis, and autophagy[14]. Cell death pathways have long been considered to function in parallel with little or no overlap. However, it is currently clear that apoptosis, necroptosis, autophagy, and ferroptosis are tightly connected and can cross-regulation each other. Gao et al. (2016), found that during ferroptosis ferritin is actively degraded *via* an autophagy pathway and the iron is released from ferritin to actively promote ferroptosis and hence he demonstrated that autophagy is important for ferroptosis initiation. Hou et al. also demonstrated experimentally that autophagy promotes ferroptosis by degrading ferritin in fibroblasts and cancer cells. And the erastin-induced ferroptosis could be inhibited by Atg5 (autophagy-related 5) and Atg7 knockouts or knockdowns, which resulted in lower intracellular ferrous iron levels and reduced lipid peroxidation (Hou et al., 2016). Briefly, modes of cell death following ICH are varied and overlapping. The mechanisms involved need to be supported by more research. Here will show the overview and comparison of different neuronal cell death types: apoptosis, necroptosis, and autophagy. Each type, along with its characteristics and mechanisms, and their potential roles in brain damage after ICH, are discussed below and are compared with the corresponding features of ferroptosis (Table 1).

**TABLE 1:** The main feature of apoptosis, necroptosis, autophagy, ferroptosis.

Type of cell death	Morphological feature	Regulators	Relationship with ferroptosis
Apoptosis	Plasma membrane blebbing, exposure of membrane phosphatidylserine, cellular and nuclear volume reduction. Nuclear shrink, nuclear fragmentation, chromatin condensation and margination	Bax, Bak, p53, Bcl-2, Bcl-XL	Ferroptosis inhibit apoptosis through the JNK signaling pathway activity
Necroptosis	Rupture of plasma membrane. Organelle swelling. Moderate chromatin condensation	RIP1/3, MLKL	Ferroptosis is always accompanied by necroptosis. NADPH might be a link between them
Autophagy	Formation of double-membraned autolysosomes	PI3K-AKT-mTOR, MAPK-ERK1/2-mTOR signal pathway	Autophagy regulates intracellular iron homeostasis and ROS synthesis to promote ferroptosis
Ferroptosis	The cell membrane did not rupture and blisters. Mitochondrial crests are reduced or disappeared, and the outer mitochondrial membrane ruptures. Normal nuclear size and chromatin	NOX, GPX4, p53, HO-1 DHODH, FSP1, BH4, GOT1, NRF2	

### 4.1. Ferroptosis and apoptosis

Apoptosis is an active process that is subject to strict gene activation, expression, and regulation, demarcated by permeabilization of the mitochondrial outer membrane and promoted by executioner caspases (Sekerdag et al., 2018). Studies on the regulation of apoptosis after ICH have been conducted earlier. In Young et al. (1989) reported that leukocytes infiltrating the brain in ICH can release harmful substances, such as proteolytic and oxidizing agents as well as cytokines, which can injure or kill cells through caspase-dependent or independent pathways (Zhang et al., 2022c). In a rabbit ICH model, the levels of active caspase-3, Fas, FasL, and active caspase-8 were upregulated in neurons near the hematoma driving neuronal apoptosis after ICH (Deng et al., 2018). Wang et al. (2022) reported that histone deacetylase 6 (HDAC6) inhibition protects against brain injury post-ICH by reducing neuron apoptosis and apoptosis-related protein expression levels by acetylation of malate dehydrogenase 1 (MDH1). Studies of tumors have shown that the ferroptosis inducer erastin activates the p53-dependent CHOP/PUMA axis and increases sensitivity to apoptosis induced by the tumor necrosis factor-related apoptosis-inducing ligand (TRAIL) (Hong et al., 2018). Ferroptosis has been shown to inhibit apoptosis through the JNK signaling pathway activity (Liu et al., 2012). Thus, there are some crosstalk between ferroptosis and apoptosis.

### 4.2. Ferroptosis and necroptosis

Necroptosis combines both necrosis and apoptosis, hence the term necroptosis (Vanden Berghe et al., 2014). It is a regulated form of necrotic cell death mediated by receptor-interacting kinase 1 (RIPK1), receptor-interacting protein kinase 3 (RIPK3), and mixed lineage kinase domain-like (MLKL) (Conrad et al., 2016; Cao et al., 2022). RIPK1 activates RIPK3 and thereby recruits MLKL at the cell membrane, which causes membrane rupture and eventually triggers necroptosis (Samson et al., 2021; Gupta et al., 2022). Necroptosis is involved in cell death associated with ICH (Galluzzi et al., 2018). Neurovascular injury and related hemolysis of extravasated erythrocytes post-ICH producing hemoglobin degradation metabolites may trigger the neuroinflammatory response of surrounding astroglia resulting in activation of the necroptotic pathway. Meanwhile, Necrostatin-1, a specific RIPK1 inhibitor, has been shown to reduce cell death, hematoma volume, and neurobehavioral outcomes in a mouse model of ICH (Gupta et al., 2022). Numerous reports have suggested that ferroptosis is always accompanied by necroptosis (Lv et al., 2021). The major ultrastructural characteristics of hemin-induced neuron death are related to ferroptosis and not necroptosis. In contrast, molecular marker levels of both ferroptosis (ferric iron, GSH, and GPX4) and necroptosis (MLKL and RIPK3) may increase after ICH. NADPH might be a link between ferroptosis and necroptosis (Hou et al., 2019). However, such studies regarding ICH are lacking (Lin et al., 2016; Newton et al., 2016; Minagawa et al., 2020; Chen et al., 2022b).

### 4.3. Ferroptosis and autophagy

Some research has revealed the important role of autophagy in ferroptosis, especially selective types of autophagy (e.g., ferritinophagy, lipophagy, clockophagy, and chaperone-mediated autophagy) (Liu et al., 2020). Ferritinophagy is the process of autophagic degradation of the iron storage protein ferritin, which is critical for the regulation of cellular iron levels. ferritinophagy promotes ferroptosis by releasing free iron from ferritin. Inhibition of Ferritinophagy inhibits ferritin degradation and therefore reduces free iron levels and thus limits subsequent oxidative injury during ferroptosis (Gao et al., 2016; Hou et al., 2016). Moreover, deficient ferritinophagy may increase the activity of iron-responsive element binding protein 2 (IREB2/IRP2) to promote ferroptosis (Dixon et al., 2012). According to Gao et al. (2016) autophagy regulates intracellular iron homeostasis and ROS synthesis to promote ferroptosis. *In vitro* experiments showed that Erastin, a synthetic small-molecule compound, which induces ferroptosis and activates autophagy, led to intracellular ferritin degradation to further increase the level of intracellular iron ions through autophagy, resulting in rapid accumulation of intracellular ROS, which promote ferroptosis. Hou et al. (2016) also demonstrated that the activation of autophagy further promoted ferroptosis by degrading ferritin in tumor cells. Suppressing autophagy is one of the ways to inhibit ferroptosis. Lipophagy, the autophagic digestion of lipid droplets can release free fatty acids. The level of lipid droplets is negatively related to oxidative stress-induced ferroptosis (Cho et al., 2022). Increased lipid droplet formation suppresses RSL3-induced ferroptosis in hepatocytes (Cho et al., 2022). In contrast, increased lipophagy promotes lipid droplet degradation and therefore increases lipid peroxidation-mediated ferroptosis (Cho et al., 2022). Chaperone-mediated autophagy (CMA) is a type of selective autophagy that uses molecular chaperones to deliver certain cytosolic proteins to lysosomes for degradation based on the recognition of specific amino acid sequences. ER stress-associated molecular chaperone, can limit erastin-induced GPX4 degradation and therefore protects against ferroptosis in pancreatic cancer cells (Zhu et al., 2017). These findings establish a model of interaction between CMA and autophagy to determine GPX4 protein stability in ferroptosis. In brief, ferroptosis and autophagy are inseparable and both contribute to neuronal death in ICH. Understanding the mechanism of autophagy and inhibiting it is one of the ways to inhibit neuronal ferroptosis.

## 5. Therapeutic application

**FIGURE 2:**
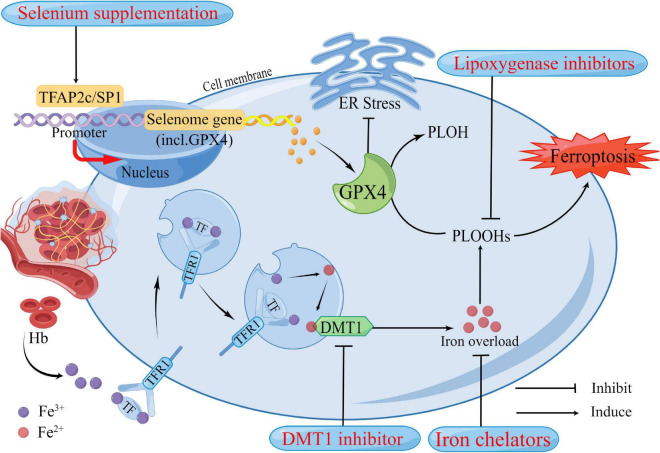
Potential therapeutic strategies based on neuronal ferroptosis after Intracerebral hemorrhage (ICH). Several regulators in the ferroptosis pathway are highlighted in the treatment of ICH. These regulators have been confirmed to play roles in ferroptosis. After ICH, in addition to the primary brain injury caused by the hematoma compressing the surrounding brain tissue, Hb, iron, and other neurotoxic substances released by the hematoma also contribute to the increase of reactive oxygen species (ROS), resulting in ferroptosis and cause secondary brain injury. DMT1 and Iron chelators could reduce iron overload from two aspects, respectively. GPX4 acts as an antioxidant that inhibits ER stress and reduces lipid peroxides in cells to harmless PLOH to inhibit ferroptosis. It has been well established that GPX4 deficiency causes neuronal ferroptosis after ICH. Selenium supplementation augments GPX4 and other genes in this transcriptional program, the selenome, *via* coordinated activation of the transcription factors TFAP2c and Sp1 to protect neurons. Hb, hemoglobin; Fe3+, ferric iron; Fe2+, ferrous iron; TF, transferrin; TFR1, transferrin receptor 1; DMT1, divalent metal transporter 1; Gpx4, glutathione peroxidase 4; PLOH, phospholipid alcohol; PLOOHs, phospholipid hydroperoxides; ER, endoplasmic reticulum.

Although the efficacy of medical interventions targeting pathological pathways of ICH has been verified in several preclinical studies, their promise has not translated to clinical trials in patients with ICH (Jin et al., 2021). Further efforts are needed to improve these limited medicinal approaches, mitigate neuronal death, and facilitate functional recovery during and after ICH. Ferroptosis has been shown to mediate the damage processes in patients with ICH (Li et al., 2020). Many previously reported neuroprotectants that showed protective effects in ICH models and patients were validated as ferroptosis inhibitors recently. Here we summarized the therapeutic targets of inhibitors of ferroptosis in ICH models (Figure 2 and Table 2).

**TABLE 2:** Reagent associated with ferroptosis.

Reagent	Target/function	Impact on ferroptosis	Mechanism
Selenium	Selenoproteins, GPX4	Stimulates the expression of the selenoproteins, such as antioxidant GPX4	GPX4 availability
DFX	Iron	Function as iron chelator, depletes iron, and prevent iron-dependent lipid peroxidation	Reduced iron overload
VK-28	Iron	Function as iron chelator, depletes iron, and prevent iron-dependent lipid peroxidation	Reduced iron overload
Deferiprone	Iron	Function as iron chelator, depletes iron, and prevent iron-dependent lipid peroxidation	Reduced iron overload
LOX inhibitors	Lipid peroxidation	Inhibits cytosolic ROS production and blocks lipid peroxidation	Radical trapping
Ebselen (DMT1 inhibitors)	Iron	Reduced the iron ion transport activity of DMT1 and prevents iron-dependent lipid peroxidation	Reduced iron overload

### 5.1. Selenium supplementation

Neurons respond to ferroptosis stimuli by induction of selenoproteins, including antioxidant GPX4. A single dose of Se delivered into the brain drives antioxidant GPX4 expression, protects neurons, and improves behavior in an ICH model. According to Tuo et al. (2021), certain selenocompounds are selective anti-ferroptotic medications that can cross the blood-brain barrier and prevent neuronal death in ischemic stroke. Recent studies demonstrated that selenium can drive protective transcriptional responses, including the transcriptional activators TFAP2c and Sp1, to upregulate GPX4 and suppress ferroptosis (Alim et al., 2019). Pharmacological Se supplementation effectively inhibits GPX4-dependent ferroptosis. The inhibition of ferroptosis and neuronal protection of selenium *via* transcriptional regulation have been verified in mouse models of ICH and ischemic stroke (Alim et al., 2019). *In vitro* and *in vivo* results highlight the potential of the pharmacological administration of selenium for the treatment of both hemorrhagic and ischemic stroke. It is also noteworthy that the Tat SelPep (a peptide that can increase GPX4 expression in the brain) can overcome the narrow therapeutic window of direct intracerebroventricular injections of sodium selenite, providing a novel strategy to deliver selenium with minimal toxicity (Alim et al., 2019).

### 5.2. Iron chelators

Intracerebral hemorrhage leads to iron overload and the upregulation of iron-handling proteins, resulting in a brain injury that can be reduced by DFX, an iron chelator, indicating that iron imbalance is an essential initiator of ferroptosis and can provide new insights into the neuroprotective activity of iron chelators (Xue et al., 2022). DFX inhibits the overactivation of microglia by forming an iron amine chelate with iron ions around the hematoma, preventing iron ions from providing electrons to oxygen to form ROS (Dixon and Stockwell, 2014; Li et al., 2017b). This processing alleviates cerebral edema, neurological deficit, and brain atrophy after ICH in rats (Selim et al., 2019). Several iron chelators have been developed. DFX was approved by the FDA in 1968 as an iron chelator that concentrates in the brain following subcutaneous injection. By sequestering nonheme iron, DFX effectively diminishes hydroxyl radical formation and reduces brain damage after subarachnoid hemorrhage (SAH) (Pandya et al., 2021). In addition to SAH, studies have reported favorable effects of DFX in various hemorrhage models, including reduced iron overload, attenuated brain–blood barrier (BBB) disruption, reduced dendritic and white matter damage, improved neurological behavior, and lower rates of mortality (Selim et al., 2019). However, according to Selim et al. (2019), the Intracerebral Hemorrhage Deferoxamine (i-DEF) trial failed to demonstrate that using DFX to treat ICH patients was sufficient enough and further research is needed to determine its effectiveness. Compared with DFX, VK-28 has a greater advantage in that it can penetrate the intact BBB which is a more effective and safer advantage. Therefore, VK-28 may act at lower concentrations in the brain, making it more suitable for clinical (Li et al., 2017a). Deferiprone, an iron chelator that can cross the blood-brain barrier, is utilized for transfusion-dependent thalassemia as well as in Parkinson’s disease clinical trials (Devos et al., 2022). These results may stimulate further development of iron chelators for ICH treatment.

### 5.3. Lipoxygenase inhibitors

Lipoxygenases inhibitors with radical trapping can function as terminators of the radical chain reactions of lipid autoxidation to inhibit ferroptosis (Poon et al., 2021). For LOX inhibitors that lack radical trapping ability, those targeting 15-LOX-1, exhibit a degree of anti-ferroptosis activity (Kagan et al., 2017). 15-LOX-1 has been regarded as a potent target for stroke treatment. Among the six LOXs isoforms (15-LOX-1, 15-LOX-2, 12S-LOX, 12R-LOX, eLOX3, and 5-LOX), 15-LOX-1 levels increase under pathological conditions in both human and mice following stroke (Yigitkanli et al., 2013; Watanabe et al., 2022). Moreover, 15-LOX-1-KO mice exhibited a protective ability against ischemic injury in several experimental stroke models (Shen et al., 2020a), highlighting the benefits of inhibiting 15-LOX-1 during stroke treatment. Targeting 15-LOX-1 during both ischemic and hemorrhagic stroke treatment showed effective and potent neuroprotective activity in several mouse models (Yigitkanli et al., 2013; Shen et al., 2020b). 15-LOX-1 inhibitors lacking radical-trapping activity might block ferroptosis by directly inhibiting the complexes. This provides a novel direction for the future development of such inhibitors.

### 5.4. DMT1 inhibitor

Divalent metal transporter 1 is a divalent metal ion transporter and is the only protein that transports ferrous iron from endosomes into the cytosol (Gao et al., 2015). In endosomes, upon release of ferric iron from transferrin following acidification, ferric iron is reduced to ferrous iron by a specific reductase and then ferrous iron is pumped in the cytosol by DMT1. After ICH, the expression of DMT1 is significantly increased. Ferrous ions induce the formation of excessive ROS and LPO, which are important factors causing ferroptosis in nerve cells (Nogueira et al., 2021). Pretreatment with the DMT1 inhibitor, ebselen, significantly reduced the iron ion transport activity of DMT1 and inhibited the production of ROS (Cheng et al., 2022). Research demonstrated that ebselen further attenuated DMT1 by inhibiting ferroptosis of neuronal cells after SAH in rats. At present, it is necessary to further strengthen the study of ebselen in cerebral hemorrhage.

## 6. Conclusion and perspectives

In recent years, the study of ferroptosis has gradually increased, and it is significant in exploring the direction of treatment and intervention for ICH. Ferroptosis is considered to be a form of regulated necrosis, which is strictly controlled at multiple levels (Stockwell et al., 2020; Chen et al., 2021). In general, ferroptosis is closely related to the intracellular iron ion, GSH, LPO, and so on factors. Selenium, iron chelators, lipoxygenase inhibitors, and DMT1 inhibitors can be used to inhibit cellular ferroptosis after ICH. It is expected to provide a new direction for the clinical treatment of ICH. Ferroptosis has more probing value in brain protection and improving neurologic function after ICH. However, more in-depth research is needed on how to translate these basic research results into clinical applications and reduce associated adverse effects.

In this review, we explored and summarized the modes of cell death after ICH, including apoptosis, autophagy, necroptosis, and ferroptosis. However, whether there is a sequential, synergistic, or other relationship between those modes of cell death is unknown and many mechanisms and regulatory factors of ferroptosis remain undiscovered. We discovered the existence of ferroptosis in neuronal cells following ICH by reviewing the literature, and also found some pathways and factors involved in regulating ferroptosis. But which plays a major role in the ferroptosis of neuronal cells after ICH, and are there other pathways of regulation? Many doubts remain to be resolved. In summary, although there are a large number of regulators that directly or indirectly affect the iron accumulation and lipid peroxidation to regulate ferroptosis post-ICH, there are still many questions that have not been answered. Further functional investigations into the complicated machinery and regulation of ferroptosis will provide a new way to effectively treat neuronal death after ICH.

## Author contributions

YZ determined the structure of the review. YC selected the references and contributed to the writing. WX contributed to the revision and finalization of the manuscript. SL prepared the all figures. All authors contributed to the article and approved the submitted version.
